# Peptide-based inhibitors of protein–protein interactions: biophysical, structural and cellular consequences of introducing a constraint

**DOI:** 10.1039/d1sc00165e

**Published:** 2021-03-25

**Authors:** Hongshuang Wang, Robert S. Dawber, Peiyu Zhang, Martin Walko, Andrew J. Wilson, Xiaohui Wang

**Affiliations:** Laboratory of Chemical Biology, Changchun Institute of Applied Chemistry, Chinese Academy of Sciences 5625 Renmin St. Changchun 130022 Jilin China xiaohui.wang@ciac.ac.cn; State Key Laboratory of Pharmaceutical Biotechnology, Nanjing University Nanjing 210023 Jiangsu China; School of Chemistry, University of Leeds Woodhouse Lane Leeds LS2 9JT UK a.j.wilson@leeds.ac.uk; Astbury Centre for Structural Molecular Biology, University of Leeds Woodhouse Lane Leeds LS2 9JT UK; Department of Applied Chemistry and Engineering, University of Science and Technology of China Hefei 230026 China

## Abstract

Protein–protein interactions (PPIs) are implicated in the majority of cellular processes by enabling and regulating the function of individual proteins. Thus, PPIs represent high-value, but challenging targets for therapeutic intervention. The development of constrained peptides represents an emerging strategy to generate peptide-based PPI inhibitors, typically mediated by α-helices. The approach can confer significant benefits including enhanced affinity, stability and cellular penetration and is ingrained in the premise that pre-organization simultaneously pays the entropic cost of binding, prevents a peptide from adopting a protease compliant β-strand conformation and shields the hydrophilic amides from the hydrophobic membrane. This conceptual blueprint for the empirical design of peptide-based PPI inhibitors is an exciting and potentially lucrative way to effect successful PPI inhibitor drug-discovery. However, a plethora of more subtle effects may arise from the introduction of a constraint that include changes to binding dynamics, the mode of recognition and molecular properties. In this review, we summarise the influence of inserting constraints on biophysical, conformational, structural and cellular behaviour across a range of constraining chemistries and targets, to highlight the tremendous success that has been achieved with constrained peptides alongside emerging design opportunities and challenges.

## Abstract 2

This review summarizes the influence of inserting constraints on biophysical, conformational, structural and cellular behaviour for peptides targeting α-helix mediated protein–protein interactions.

## Graphical abstract

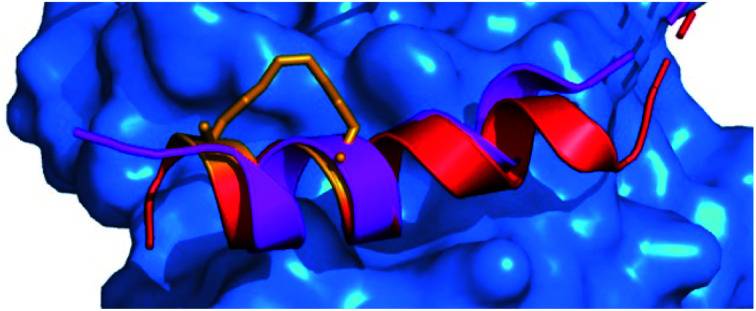

## 1 Introduction

Protein–protein interactions (PPIs) mediate virtually all biological processes and are associated with many diseases. PPIs have historically represented challenging targets for competitive (orthosteric) inhibitor discovery; they typically involve interaction of comparatively large and less featured protein surfaces, in comparison to established drug targets.^1^

The α-helix has been shown to have a relatively high prevalence in the human PPI interactome^2,3^ and represents a generic pharmacophore for ligand design.^4^ As a result, α-helix mediated PPIs have attracted significant attention for the development of selective probes and drug candidates to meet a plethora of unmet therapeutic needs.^5^ At such PPI interfaces, the α-helix of one protein is bound within a groove on the binding partner. A significant number of such interactions involve short peptide motifs;^6^ these are typically located within intrinsically disordered regions (IDRs),^7–9^ thus the α-helix is transiently stabilized on formation of the PPI. Crystallographic and structural analyses of these interfaces can facilitate the development of peptides (or judiciously designed small molecules) that mimic the helix both topologically and/or topographically; such mimetics (termed ‘peptidomimetics’) offer a promising starting point in the development of PPI inhibitors and have led to clinical candidates.^10^ Constrained peptides represent a branch of peptidomimetics that have emerged as powerful tools for perturbing PPIs; chemically constraining (or ‘stapling’) a peptide in its bioactive α-helical conformation has been reported to confer numerous benefits such as enhanced protease resistance, stability in cells, increased cellular uptake and improved biophysical properties in comparison to wild-type sequences.^11–13^

Just as the position of a constraint within a sequence has effects on peptide helicity, the nature of chemical linker can also influence its structure and function. Since the pioneering work of Grubbs^14^ and Verdine^15^ to develop hydrocarbon stapling, an extensive toolkit has been elaborated to chemically constrain peptides.^16,17^ These tools include: disulfide bonds, lactam bridges, hydrogen bond surrogates, alkanediyl tethers, bridges from thiol–ene coupling, triazole-staples from “click” chemistry and supramolecular approaches, with a number developed to allow further functionalization *via* the constraining linker.^17–25^ With such synthetic diversity on offer, it is reasonable to ask how can one predict which staple type will be most suitable? Different chemical linkers induce helicity in a given sequence to different extents and, when constraining a peptide in its bioactive α-helical conformation, the optimal flexibility is target dependent.^26^

In this review, we provide an overview of constrained peptides demonstrating non-classical biophysical or structural behaviour. We focus predominantly on α-helix mediated PPIs although note the approach has been broadened to other classes of PPIs.^27,28^ We collate recent and unusual observations that highlight the different influences staple types can have on both peptide conformation and target binding. Finally, we discuss the most recent literature on mechanisms of cell penetration and studies to ascertain the characteristics of constrained peptides that enhance cellular uptake. Ultimately, we intend that this review will aid the rational design and development of highly potent and cell penetrating constrained peptides as PPI inhibitors.

## 2 Enhancing protein binding affinity

Peptides that adopt an α-helical conformation as a part of their parent protein are often disordered in solution. Upon peptide-protein binding, α-helix formation occurs through a nucleation, followed by propagation mechanism.^29^ The entropic ‘cost’ associated with helix folding may thus impair target protein binding. Introduction of a chemical linker to constrain (or ‘staple’) the peptide in its bioactive α-helical conformation might be anticipated to overcome this entropic cost and enhance target protein binding affinity, however this is not always the case and justifies application of a more nuanced appreciation of ligand–receptor binding theory.

### 2.1 The importance of binding kinetics, thermodynamics and mechanism

**Fig. 1:**
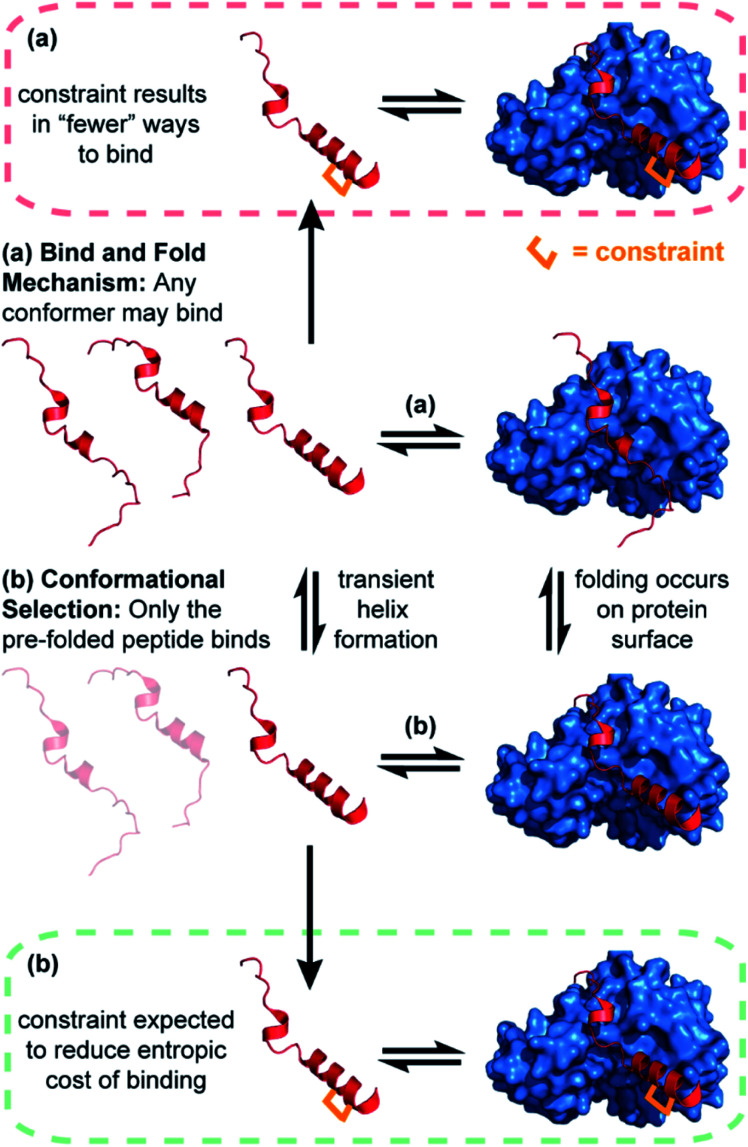
Schematics depicting different binding mechanisms for peptide–protein interactions; (a) bind and fold (b) conformational selection. Where binding is conformational-dependent (b), the introduction of a constraint is anticipated to enhance binding affinity (green dashed line). Where a bind and fold mechanism occurs (a), the introduction of a constraint may have no effect on, or reduce, binding affinity (red dashed line).

The idea that pre-organization of a peptide into its bound bioactive conformation will enhance target protein binding affinity is based on various assumptions. One is that the target protein only recognises the peptide when it forms a helical structure. Where binding proceeds by such conformational selection,^30^ constraining a peptide can be expected to increase the equilibrium concentration of the bioactive conformer, resulting in a more efficient interaction with its target (Fig. 1b). In contrast, this may not be expected for a peptide–protein interaction where the unstructured peptide is also recognised by the target. Where a less structured peptide is recognised and folds on a binding surface, it is best described as a “bind and fold” mechanism.^31^ In these cases, a constrained and pre-organised peptide might be expected to have limited “ways to bind” (Fig. 1a), resulting in a slower rate of binding and unbinding with little or a negative impact on affinity.^32^

Similarly, the impact of pre-organization on both the enthalpy and entropy of binding warrants consideration.^33,34^ When a peptide folds, enthalpy associated with folding includes contributions from backbone and side chain hydrogen-bonding, and other electrostatic contributions, together with enthalpic contributions from changes in solvation.^35^ Similarly, the total entropy of folding includes solvent reorganisation, changes in rotational–translational freedom and conformational ordering of the peptide.^34^ For the example of an unbound peptide in solution, the unconstrained variant will be more disordered whilst water molecules will be ordered around exposed backbone amides in contrast to the constrained peptide.^35^ Upon folding, the unconstrained peptide will co-operatively gain new hydrogen-bonds between backbone amides while the constrained peptide will not gain these enthalpically favourable contributions to the same extent, yet less impact from the entropically favourable release of water can be anticipated.^36^ Thus, the opposing entropic and enthalpic contributions (enthalpy–entropy compensation) often result in only marginal increase in stability of a folded form *i.e.* the net Δ*G* of folding may be comparable to the magnitude of a single non-covalent interaction (*i.e.* ∼<5 kJ mol^−1^) and this might be considered the maximum accessible gain arising from pre-organization (in the absence of additional interactions being introduced between constrained peptide and target protein; see later). Such a framework ultimately represents an oversimplification; making the assumption that constraining a peptide changes the energetic state relative to the wild-type sequence is not valid as the sequences are different. Constraining a peptide simply increases the stability/energy of its unfolded form.

In a study to explore how pre-organization of BH3-family peptides affects their inhibitory potency of the BH3/BCL2-family PPIs,^37^ specifically BID and BIM BH3 domains with MCL-1 and BCL-x_L_, Miles *et al.* found that whilst the introduction of a hydrocarbon constraint increased the population of the bioactive α-helical conformation of the peptides in solution, this did not enhance their potency.^35^ In fact, some of the constrained peptides exhibited a significant loss of potency. Co-crystal structures of the constrained BH3 peptides bound to the BCl-2 family proteins showed that the constraint induced no significant differences in the orientation of hot-spot side chains or registry of the peptide, nor did the constraint overtly introduce a steric clash with the target protein. Thus, the unexpected drop in potency could not be explained by the static structure of the bound complexes. Surface plasmon resonance (SPR) assays revealed that the rates of binding and unbinding differed between wild-type and constrained sequences; in all cases, the introduction of a hydrocarbon constraint resulted in a significant decrease in both on and off rates consistent with a bind and fold mechanism of binding. Van't Hoff analyses of fluorescence anisotropy direct binding experiments allowed the contributions of enthalpy and entropy to the interaction to be determined; these data revealed that the entropic cost of binding was indeed reduced for the constrained peptide. However, the favourable change in entropy was compensated for by an opposing change in the enthalpic contribution to binding.^35^ These observations are consistent with what might be expected when constraining a peptide that interacts with its target through a bind and fold mechanism of interaction.

Ochsenbein and co-workers also observed enthalpy–entropy compensation in studies on the histone H3/ASF1 (anti-silencing function 1) interaction.^38^ In this work, variant H3 peptides (res 118–135) bearing *i*, *i* + 4 hydrocarbon staples in multiple positions within the sequence were investigated.^39^ In addition to highlighting the above discussed limitations in correlating pre-organization with binding affinity, biophysical analyses also revealed that promotion of α-helix formation is dependent on the position at which the constraint is introduced in the sequence. This is unsurprising, since different peptide sequences have different propensities for α-helix formation and replacing different amino acids in a native sequence with unnatural amino acids (used to incorporate a constraint) will influence this natural propensity to differing extents.

Important insight into the effects of constraining peptides^40^ has been obtained using stapled peptide ligands for the Eukaryotic translation initiation factor 4E (eIF4E); a protein that regulates cap-dependent mRNA translation *via* interactions with competing binding partners, 4E-BP1 and eIF4G.^41,42^ Both 4E-BP1 and eIF4G bind to the same canonical groove of eIF4E and bind *via* intrinsically disordered regions (IDRs) which form α-helical motifs when bound. Using molecular dynamics (MD) simulations it was shown that hydrocarbon constrained eIF4G peptides experienced changes in structural dynamics when bound or unbound to eIF4E. Although stabilization of the unbound peptide in a helical conformation could be readily achieved, binding could also be impeded by favouring metastable conformations that had to change on target binding to allow key side chains to adopt the orientation required for molecular recognition. These analyses were supported by experimental and structural studies, highlighting the importance of stabilizing solution conformations that match the bound conformation. Rational design subsequently led to stapled-peptides with enhanced target residence time targeting an unexploited patch on the surface of eIF4E.^43^ Similar observations were made by Gallagher *et al.*^44^ Initially, the group measured the helical propensities of the linear wild-type 4E-BP1 and eIF4G peptides and found that the 4E-BP1 sequence exhibits greater helicity in solution than the eIF4G. SPR was used to profile the temperature- and salt-dependence of the peptide–protein interactions; whilst eIF4G binding was found to depend on electrostatic contributions and vary in binding kinetics with temperature, 4E-BP1 was relatively unaffected. The data indicated that although 4E-BP1 and eIF4G form similar bound structures, the two peptides adopt distinct binding mechanisms with the 4E-BP1 peptide exhibiting a greater complementarity for eIF4E and forming a more stable bound complex.^44^ By comparing the effects of hydrocarbon stapling on the two sequences, Garner and co-workers demonstrated a correlation of the linear 4E-BP1 and eIF4G binding kinetics with the corresponding stapled peptide properties. Whilst stapling the more complementary 4E-BP1 sequence improved affinity and bioactivity (indicating conformational selection operates for 4E-BP1), stapling eIF4G produced a nonhelical macrocyclic peptide with poorer affinity for eIF4E than its linear counterpart. Therefore, this study again emphasized that the analysis of binding mechanism and kinetics can play a crucial role in understanding whether an IDR peptide will benefit from the introduction of a conformational constraint.

**Fig. 2:**
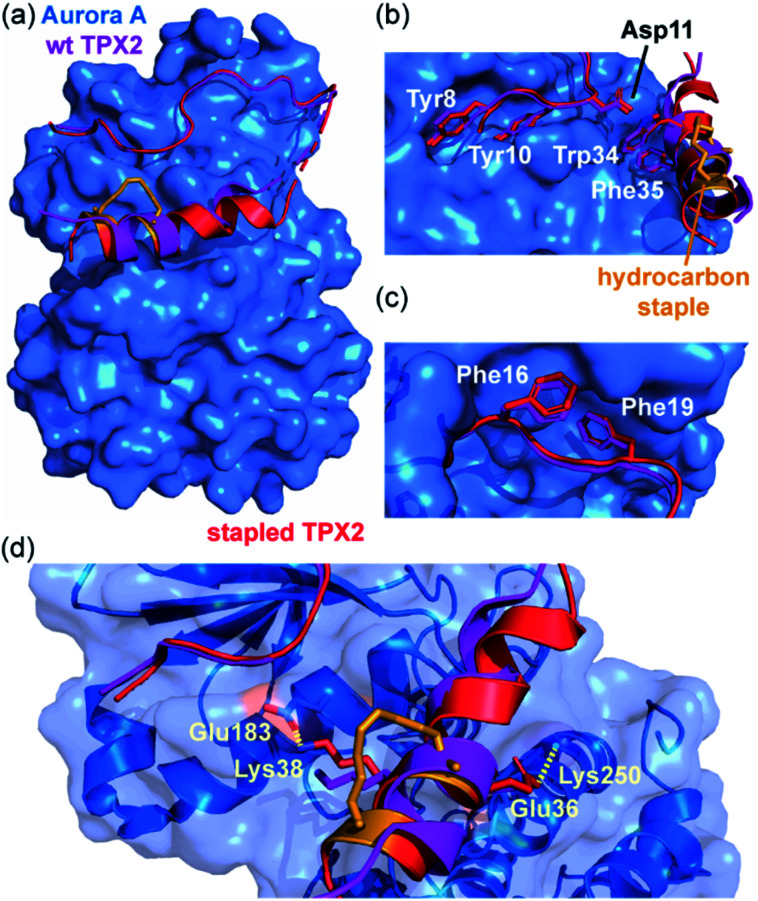
Comparison of unconstrained/constrained Aurora-A bound TPX2 peptides; (a) view of Aurora-A (blue) bound to stapled TPX2 (red, hydrocarbon staple: orange, PDB: 5LXM) with native TPX2 (purple, PDB: 1OL5) overlaid. TPX2 residues known to be crucial for binding to Aurora-A are shown as sticks to highlight the conserved binding mode between stapled and native TPX2; (b) Tyr8, Tyr10, Asp11, Trp34 and Phe35; (c) Phe16 and Phe19; (d) orientations of residues Lys38 and Glu36 differ, promoting additional electrostatic interactions (dashed lines) between peptide and Aurora-A.^45^

Jamieson and co-workers adopted a constrained peptide approach to target the α-helix mediated PPI of mitotic kinase Aurora-A with microtubule-associated protein TPX2.^45^ In this work, a hydrocarbon stapled TPX2 peptide demonstrated improved binding affinity for Aurora-A and was even shown to mimic the function of TPX2 in activating autophosphorylation of the kinase. However, a closer inspection of the thermodynamic determinants of binding, as measured by Isothermal Titration Calorimetry (ITC), revealed a surprising finding; although the introduction of the constraint increased the population of the bioactive α-helix and enhanced the binding affinity, the entropy of binding for the constrained peptide was more unfavourable when compared to the wild-type. In concordance, the constrained peptide had a more favourable enthalpy of binding, suggesting it made more favourable interactions with the Aurora-A protein when bound. This hypothesis was later confirmed *via* crystallographic analysis, by superposing the existing structure of Aurora-A in complex with native TPX2 (PDB: 1OL5) onto a crystal structure of the bound stapled TPX2 peptide (PDB: 5LXM; Fig. 2a). Although the hydrocarbon staple itself makes no contacts with the Aurora-A surface and the crucial hot-spot residues remain in almost identical orientations between the two structures (Fig. 2b and c), the staple extends the length of the helix by an additional turn and alters the conformation of the bound helix, allowing two additional charged side chains, Glu36^TPX2^ and Lys38^TPX2^, to contribute to hydrogen-bonding interactions with Aurora-A (Fig. 2d). Thus the less favourable entropy can be accounted for by the requirement to form a longer more ordered helix so as to make the additional enthalpically favourable non-covalent contacts.

Hetherington *et al.* prepared and investigated a series of constrained peptides to target HIF-1α/p300;^46^ a PPI which regulates oxygen levels in cells and is often hijacked by cancer to supply growing tumours with oxygen.^47–49^ Here, the peptides were constrained through reaction of dibromomaleimide with *i* and *i* + 4 Cys residues.^21,50^ One of the dibromomaleimide (DBM) stapled HIF-1α peptides demonstrated a significant enhancement of binding affinity for p300 in comparison to the unconstrained native peptide. However, contrary to expectation, the enhanced inhibition did not correlate with an increase in α-helicity as shown by circular dichroism (CD) experiments. In an attempt to explain the beneficial effects of introducing the DBM staple, the group compared MD simulations of the HIF-1α variants both in solution and in complex with p300. MD simulations indicated that both the constrained and unconstrained peptides showed greater helical character in the bound state when compared to the unbound peptides, with a more dramatic increase observed for the DBM stapled peptide. Thus, the staple-induced affinity enhancement was proposed to occur as a result of stabilizing the bound state of the peptide in complex with p300 ^46^ and was supported by experimental CD difference experiments. In a similar vein, Grossmann and co-workers also observed that changes in the behaviour of bound state as a consequence of introducing a constraint are important for interaction of Exoenzyme S (Exo S) derived peptides with 14–3–3 protein although in this case, increased affinity was attributed to increased dynamics in the peptide-receptor complex.^51^

**Fig. 3:**
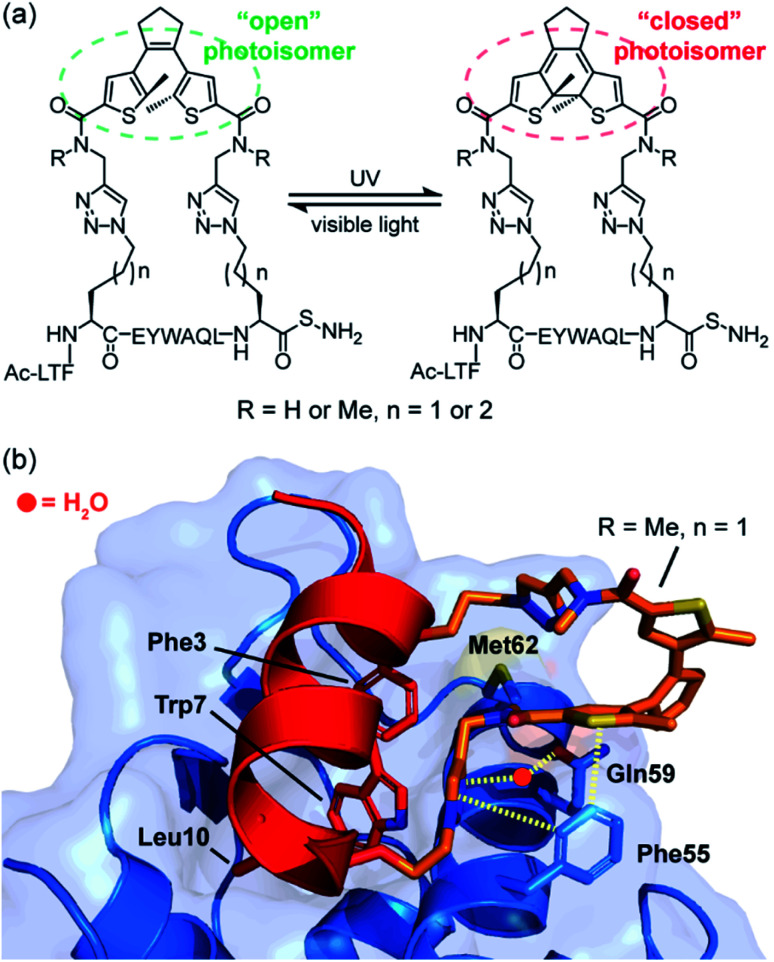
MDM2 binding peptides with photoswitchable constraints that exhibit distinct thermodynamic signatures; (a) structures of both “open” and “closed” DAE stapled photoisomers, (R = H or Me, *n* = 1 or 2) used by Spring and co-workers to target the p53/MDM2 interaction; (b) crystal structure of MDM2 (blue) in complex with the highest affinity “open” analogue (red, PDB: 6Y4Q). Residues known to be crucial for binding to MDM2 are shown as sticks: Phe3, Trp7, Leu10, with the linker shown in orange making direct or H_2_O-mediated contacts (dashed lines) with MDM2.^52^

In studies by Strizhak *et al.* a library of peptide analogues, based on a known p53/MDM2 peptide discovered by phage display,^53^ were stapled with a photoisomerizable diarylethene (DAE) moiety between the *i*, *i* + 7 residues using azide–alkyne “click” chemistry.^52^ Each analogue possessed two photoisomers, referred to as “open” or “closed”, depending on exposure to visible or UV light, respectively (Fig. 3a). Analogues also differed in linker length (*n* = 1 or 2) and/or *N*-methylation (R = H or Me). By utilizing competition assays based on tryptophan fluorescence quenching, an interesting observation was noted; analogues constrained with “open” DAE photoisomers were consistently stronger binders to MDM2 than their “closed” counterparts. Moreover, ITC measurements, performed to gain insight into the thermodynamic parameters of binding, revealed that binding of the “closed” forms were mostly enthalpy-driven, whilst the “open” isomers bound with a greater entropic contribution. Attempts to crystalize the highest affinity analogue (R = Me, *n* = 1) bound to MDM2 were successful for the “open” form (Fig. 3b). The peptide bound to MDM2 as expected, forming an α-helix, with the Phe3^p53^, Trp7^p53^ and Leu10^p53^ hot-spot residues occupying the known lipophilic pockets on the protein surface (Fig. 3b). The linker was shown to directly interact with the target protein by edge-to-face π-stacking interactions between the triazole and thiophene moieties of the linker and Phe55^MDM2^. The triazole ring also forms hydrogen bonds to a local water molecule, which, in turn, forms a hydrogen bond with Gln59^MDM2^. Although the “open” isomer has greater conformational freedom, given the structural data illustrate additional non-covalent interactions can form, the rationale for the observed greater entropic contribution to binding in comparison to the “closed” form is not fully clear. Nonetheless, this example illustrates the potential for the linker of a constrained peptide to make a contribution to molecular recognition which we discuss in greater detail in the following section.

### 2.2 Forming more contacts: direct interactions between staple and target

In designing constrained peptides for modulation of α-helix mediated PPIs, the constraint has typically been introduced on the solvent-exposed surface of the α-helix to avoid introducing a steric clash with the binding surface and/or interfering with the interaction of hot-spot residues.^54–57^ However, in a number of instances the chemical linker has been shown to interact profitably with the target protein to enhance target binding, or even to change the nature of the interaction. The ability to design constrained peptides such that the constraint makes additional, favourable contacts with the target protein could be beneficial for their development. The following examples focus largely on co-crystal structures that highlight this behaviour and point to some of the challenges in intentionally designing such constraints.

**Fig. 4:**
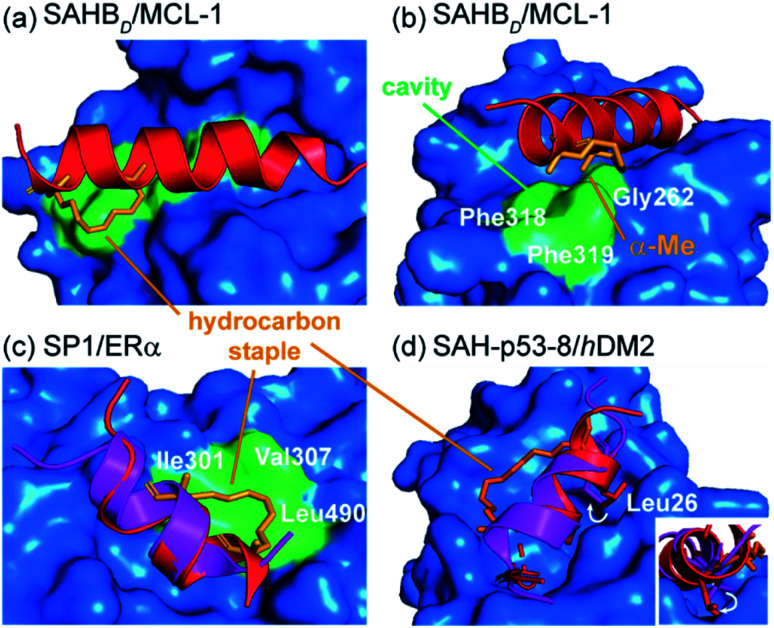
Co-crystal structures of protein-bound constrained peptides highlighting the potential for contact between constraint and protein; (a) crystal structure of the MCL-1 SAHB_D_/MCL-1 complex (PDB: 3MK8)^58^ and the hydrocarbon staple (orange) of MCL-1 SAHB_D_ makes additional hydrophobic contacts at the perimeter of the core interaction site; (b) a methyl group of the α,α-disubstituted functionality occupies a groove defined by Gly262, Phe318 and Phe319 of MCL-1 (green); (c) comparison of the ERα bound structures of the native sequence (purple, PDB: 2QGT) and the stapled peptide SP1 (red, PDB: 2YJD);^60^ (d) comparison of the *h*DM2 bound structures of the native p53 peptide (purple, PDB: 1YCR)^61^ and the stapled peptide (red, PDB: 3V3B).^62^

Walensky and co-workers screened a library of stabilized α-helix BCL-2 domains (SAHBs), and determined that the MCL-1 BH3 helix is itself an MCL-1 inhibitor.^58^ Through a combination of site-directed mutagenesis and staple scanning studies, the group developed an optimized, hydrocarbon stapled MCL-1 peptide (MCL-1 SAHB_D_). To structurally define key interactions, a crystal structure of the stapled peptide bound to MCL-1 was obtained (Fig. 4a). This crystallographic analysis revealed that the hydrocarbon staple itself makes discrete hydrophobic contacts with the perimeter of the MCL-1 binding site (Fig. 4a). Moreover, a methyl group of the disubstituted unnatural amino acid was shown to occupy a hydrophobic cavity defined by Gly262^MCL-1^, Phe318^MCL-1^ and Phe319^MCL-1^ whilst additional contacts were also evident for the aliphatic side chain (Fig. 4b). Therefore, the superior binding affinity of the optimized, hydrocarbon stapled peptide potentially derives from two contributions: firstly, enhanced α-helicity as a result of pre-organization and secondly, from additional hydrophobic contacts by the staple itself. Keating and co-workers similarly observed extensive interaction between the staple and MCL-1 in their optimization of a lead peptide for clinical development.^59^

Phillips *et al.* highlighted significant considerations for the design of constrained peptide inhibitors through the development of stapled peptides for nuclear receptors (NRs).^60^ An 11-mer stapled peptide with sequence Ac-H-S_5_-ILH-S_5_-LLQDS-NH_2_ (SP1, where S_5_ is (*S*)-2-(4-pentenyl)alanine) was designed to target the coactivator binding site of the estrogen receptor (ER), and a staple scan carried out to establish the optimal constraint. Structures of these complexes and of the peptides in isolation were studied. For the most potent stapled peptide (SP1), bound to the ligand binding domain of ERα, inclusion of the hydrocarbon staple significantly increased the helicity of the peptide as judged by CD spectroscopy and NMR. However, the crystal structure of the SP1/ERα complex revealed that the hydrophobic staple itself formed favorable contacts with the hydrophobic surface defined by Val307^ERα^, Ile310^ERα^, and Leu490^ERα^ (Fig. 4c). Comparison with the coactivator protein–peptide complex (2QGT) revealed a difference of a quarter turn of the helix, shifting the binding site residues out of register by one position. Therefore, the introduction of the hydrocarbon staple not only conformationally restrained the peptide, but induced a non-canonical mode of interaction with the potential to lead to non-specific effects.

Later, Baek *et al.* reported a crystal structure of a hydrocarbon stapled p53 peptide (SAH-p53-8)^63^ in complex with its binding partner, *h*DM2.^62^ The crystal structure revealed that the staple occupied a hydrophobic region on the rim of the p53 binding site on *h*DM2, contributing approximately 10% of the total surface contact area between peptide and protein, likely enhancing binding affinity. Stapling of the peptide imposed perfectly helical angles (−58°/−45°) on Leu26^p53^ in the structure of the constrained peptide resulting in a more helical bound-state when compared to the wild-type, unconstrained counterpart. Moreover, the resulting conformational change adjusted the Leu26^p53^ side chain orientation, leading to a stronger interaction with *h*DM2 (Fig. 4d). Similar observations were made for ATSP-7041 – a clinical candidate peptide-based inhibitor of *h*DM2 and *h*DMX developed by Aileron.^64^

**Fig. 5:**
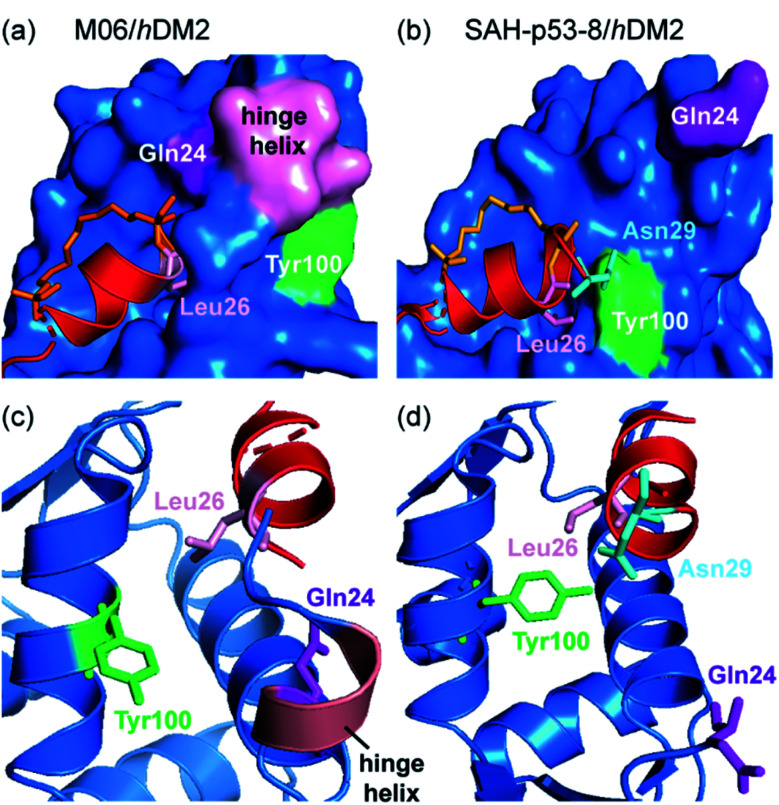
Comparison of constrained p53/*h*DM2 structures; (a) the binding site formed in the crystallographically unique M06 complex is capped by the ‘hinge’ helix of the *h*DM2 lid (PDB: 4UMN); (b) the binding pockets that form when *h*DM2 interacts with SAH-8 (PDB: 3V3B);^62^ (c) The ‘hinge’ helix caps the bottom of the pocket; (d) Tyr100 (green) adopts the ‘closed’ conformation and forms a hydrogen bond interaction with Asn29.^65^

Ghadessy and co-workers reported a crystal structure of the hydrocarbon stapled peptide M06 – a variant of one previously reported by Brown *et al.*^66^ – in complex with *h*DM2.^65^ The staple was shown to pack favourably against hydrophobic residues of *h*DM2. In addition, the M06/*h*DM2 crystal structure showed that the peptide binding groove was capped by the helical ‘hinge’ region of *h*DM2 (Fig. 5a), near to the C-terminus of the bound peptide. Comparison with the SAH-p53-8/*h*DM2 crystal structure reported by Baek *et al.*, reveals significant differences (Fig. 5b).^62^ In the M06 complex the shorter helix of M06 does not fill the *h*DM2 pocket as completely as SAH-p53-8, such that the hinge region of *h*DM2 adopts a helical conformation that caps the pocket, accommodating a tight fit of M06 (Fig. 5c). In the SAH-p53-8/*h*DM2 structure, Tyr100 of *h*DM2 points into a ‘pocket’, stabilized by a hydrogen bond interaction with Asn29^p53^. This results in occlusion of the pocket, and in turn, shields Leu26^p53^ from solvent (Fig. 5d). It is clear that this movement of the hinge can be accommodated by Tyr100^*h*DM2^ either projecting into (‘closed’ conformation) or out (‘open’ conformation) of the p53 binding site on *h*DM2. Thus even though a constraint can form interactions with the target protein, this example highlights that in doing so, it can induce conformational changes adding to the challenge of designing constraints so as to optimize non-covalent interactions with target proteins.

### 2.3 The staple structure: influence on peptide conformation and target binding

As discussed in the preceding section, the chemical staple used to constrain peptides can, in certain cases, interact directly with the target protein to enhance binding affinity. Minor alterations to the constraint, such as stereochemistry at the α-position, α-substituent groups and additional branching on the hydrocarbon *e.g.* a γ-Me group, insert-site and linker length can thus significantly impact on the behaviour of constrained peptides. Indeed, during the development of the hydrocarbon staple it was noted that small changes in linker length resulted in significant effects on the efficiency of stapling reactions and consequently the effects on peptide structure.^15^ Below we discuss several examples that showcase the diverse influence of staple structure on peptide conformation and binding.

Traditionally, incorporation of unnatural α-methyl, α-alkenyl disubstituted amino acids at *i*, *i* + 4, or *i*, *i* + 7 positions, is used to introduce hydrocarbon staples. The α-methyl group was introduced to the unnatural amino acids for its added helix-stabilizing effect.^15,67^ Yeo *et al.* explored this using the BCL-2/BH3 family PPIs as a model; comparison of a BID sequence constrained using α-methyl, α-alkenyl amino acids (BID-DM) with the α-alkenyl monosubstituted variant (BID-MM) revealed comparable helicity, resistance to proteolysis and potency for both.^68^ However, such behaviour may not hold in every case (*cf.* the role of the methyl group in recognizing MCL-1, Section 2.2 and Fig. 4b).

Grossman and co-workers recently highlighted the profound effects that α-methylation can have on binding behaviour of constrained peptides that target the trimeric nuclear transcription factor Y (NF-Y) complex.^69^ Initially, incorporation of an *i*, *i* + 4 α-methyl, α-alkenyl hydrocarbon staple was shown to reduce the binding affinity of a native, 19-residue NF-YA peptide for the NF-YB/C dimer, despite increasing the helicity of the free peptide in solution from 13% to 47%.^70^ In contrast a shorter 16-residue analogue bearing the exact same constraint had significantly higher affinity. Since the precise implications of α-methylation remain unclear, the group expanded their study to explore whether the α-methyl groups caused the loss of affinity upon peptide elongation. Switching the N-terminal disubstituted amino acid for its monosubstituted counterpart^68^ resulted in >10-fold affinity enhancement. Both NMR and CD analysis revealed that the removal of this methyl group had negligible impact on peptide structure in solution, whilst crystallographic data revealed that the methyl group did not make direct contacts with the target protein. The intriguing result was found to derive exclusively from the conformational characteristics of the bound peptides; a combination of 2D ^1^H–^1^H TOCSY and transfer-NOE NMR experiments of the constrained peptides in the presence and absence of NF-YB/C dimer revealed that the α-methyl group caused the N-terminus of the peptide to deviate considerably from its bound form.^70^ The thermodynamic parameters for binding reflect the structural differences; the fully α-methylated variant exhibited a reduced entropic cost of binding which was countered by a decrease in binding enthalpy, whereas the stronger binding mono-methylated variant had a much more unfavourable entropy of binding that was compensated by a large enhancement in binding enthalpy. Such results underscore the importance of biophysical and structural considerations in the development of potent peptidomimetic PPI inhibitors.

**Fig. 6:**
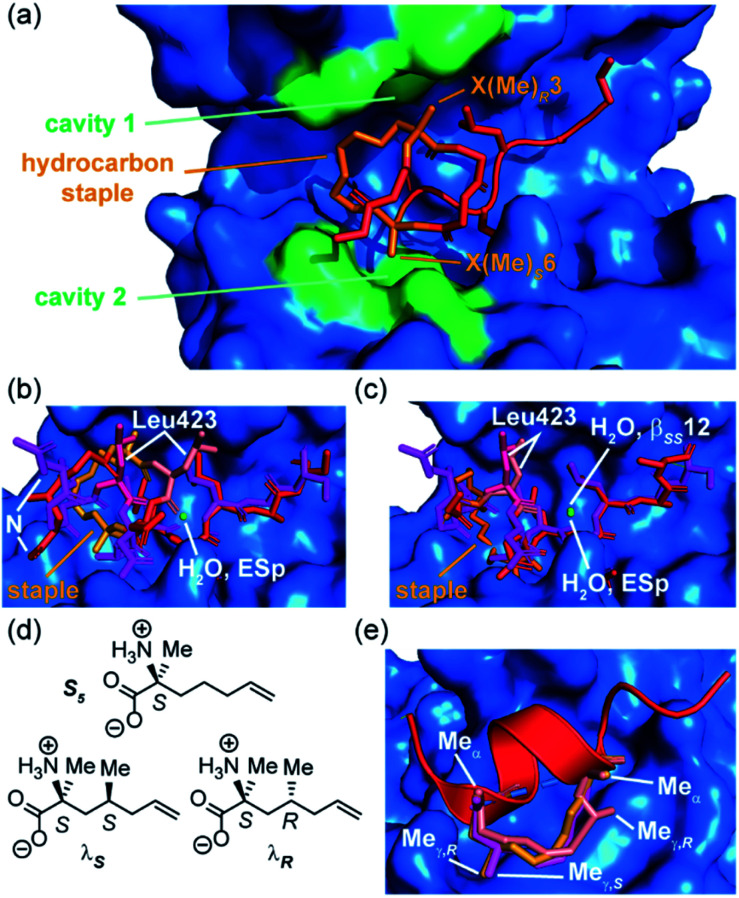
Effects of branching on staple behavior; (a) crystal structure of hydrocarbon cross-linked macrocyclic peptide (red, hydrocarbon staple: orange, PDB: 4N7Y) in complex with 14–3–3 (blue): computational analysis of the 14–3–3 surface reveals cavities 1 and 2 (green) in proximity to amino acids X(Me)_*R*_3 and X(Me)_*S*_6 that may be suitable sites through which to optimize affinity; (b) crystal structures of ESp (purple) and β_*RS*_8 (PDB: 4N7G, red) bound to 14–3–3 (PDB: 4N7Y); (c) crystal structures of ESp (purple, PDB: 4N7G) and β_*SS*_12 (red) in bound to 14–3–3 (PDB: 4N84);^28^ (d) structure of branched stapling amino acids S_5_, λ_*S*_ and λ_*R*_; (e) SRC2-SP1 (salmon, PDB: 5DXB), SRC2-SP2 (orange, PDB: 5HYR), and SRC2-SP3 (purple, PDB: 5DX3) adopt conformations to alleviate *syn*-pentane interactions between the α- and γ-methyl groups.^74^

The Grossmann group also investigated the effects of altering the smaller alkyl substituent on the α-carbon of disubstituted amino acids.^71^ The group previously reported a hydrocarbon cross-linked macrocyclic peptide, derived from the pathogenic protein ExoS, to target the protein interaction site of the human adaptor protein 14–3–3.^28^ Originally, this peptide comprised 11 key amino acids^72,73^ and contained an *R*- and an *S*-configured α-methyl, α-alkenyl amino acid at positions 3 and 6, X(Me)_*R*_3 and X(Me)_*S*_6, which were connected to form an eight membered hydrocarbon linker (Fig. 6a) and found to adopt an irregular structure when bound to human adaptor protein 14–3–3.^28^ This work highlighted the potential to exploit stapling to stabilize recognition motifs other than the α-helix. After truncation studies identified a minimal sequence which maintained binding efficiency, the group systematically replaced the α-methyl group of the disubstituted amino acids, X(Me)_*R*_3 and X(Me)_*S*_6, with either hydrogen or a more hydrophobic ethyl substituent. These alterations were applied to both disubstituted amino acids giving a total of seven variants. Interestingly, all variants with at least one hydrogen-substituent experienced a loss in binding affinity, whilst those with α-ethyl substituents were stronger binders. Whilst ethyl-modification at position 3 resulted in pronounced affinity enhancement, the same modification at position 6 had little impact; thus, the effects of introducing ethyl groups at both positions were not additive. An extensive investigation of the conformational diversity of the free peptides in solution was used to rationalize the results. MD simulations identified two predominant conformer populations of the macrocyclic peptide in solution and, crucially, the alkyl substituent was observed to play a role in biasing the conformation towards the higher affinity of the two dominant conformers. A degree of correlation with log *D* values also indicated a role of hydrophobicity consistent with the larger alkyl substituents making direct contact with the protein. A co-crystal structure of the optimized macrocyclic peptide in complex with 14–3–3 revealed that the α-ethyl substituent in position 3 was able to insert into a hydrophobic cavity on the protein surface (cavity 1) more so than the smaller α-methyl group (Fig. 6a). Thus, the minor structural alteration from α-methyl to α-ethyl had a synergistic stabilizing influence on both the free and bound peptide.^71^ Constrained peptides with different configuration and length of linkers (*i*, *i* + 3 and β_*RS*_8, β_*SS*_12) were also developed with increased affinity for 14–3–3 in comparison to the wild-type ESp sequence. Protein X-ray crystallography was employed to explain in mechanistic detail how the constraint contributed to target binding, and explain the structure–activity relationship of different linker lengths and configurations (Fig. 6b and c).^28^ The backbone in β_*RS*_8 was observed to differ significantly to that of wild-type ESp, resulting in loss of direct, as well as water-mediated, polar interactions and a dislocation of the crucial Leu423 residue. In contrast, the arrangement of the backbone in bound β_*SS*_12 was similar to that of the wild-type ESp peptide, including water mediated interactions.

To mimic interactions of branched hydrophobic side chains of leucine and isoleucine, Speltz *et al.* created unnatural amino acids that incorporate a methyl group in the γ-position (λ_*S*_, λ_*R*_) of the stapling amino acid S_5_ (Fig. 6d and e) and introduced them into a sequence derived from the ERα binding steroid receptor coactivator 2 (SR2).^74^ CD analyses of the peptides indicated that the wild-type sequence is disordered in solution, while the stapled peptide with an *S*-γ-methyl stapled amino acid adopted an α-helical conformation and exhibited significantly higher affinity for ERα. Moreover, the study found that the addition of γ-methyl groups may positively impact affinity while having a slightly negative effect on helicity, implying that constructive interactions with the surface of the receptor are potentially more important for affinity than conformational pre-organization. Further crystal structures of bound peptides containing γ-methyl groups in the *R* or *S* configuration at the *i* or *i* + 4 (Fig. 6e, SRC-SP1, SRC2-SP2 and SRC2-SP3) positions demonstrated that such modifications are not only tolerated, but allow the hydrocarbon staple to effectively mimic branched amino acid side chains.

### 2.4 Effects of type of constraint

**Fig. 7:**
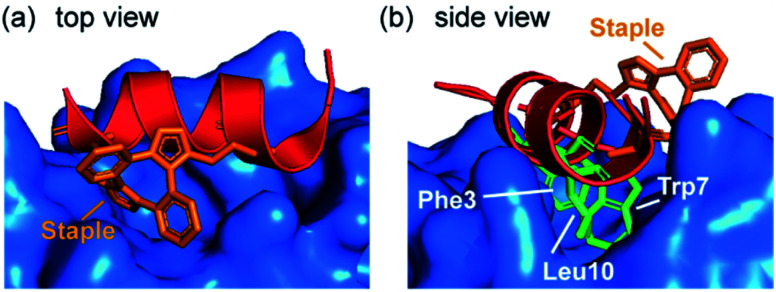
Crystal structure of bis(triazolyl) staple peptide bound to MDM2 (PDB ID: 5AFG), showing the α-helical conformation and the anti-regioconnectivity of the staple.^75^

Spring and co-workers illustrated that the ability of the constraint to make productive interactions with target proteins is not limited to hydrocarbon staples. MDM2 binding peptides based on the phage-derived PMI/PDI peptides were obtained using a strain-promoted alkyne–azide cycloaddition (SPAAC) stapling procedure developed to accelerate discovery of cell-active stapled peptides.^75^ This *in situ* stapling/screening process was used to identify a stapled peptide boasting improved proteolytic stability and nanomolar binding to its target protein, MDM2. The α-helical conformation and the anti-regioconnectivity within the staple were later confirmed by a crystal structure of the bound-peptide complex. The structure illustrated that the bis(triazolyl) constrained peptide oriented the binding triad (Phe3^p53^, Trp7^p53^, Leu10^p53^) correctly to engage the p53 binding site on MDM2 (Fig. 7) and formed interactions with the protein.

**Fig. 8:**
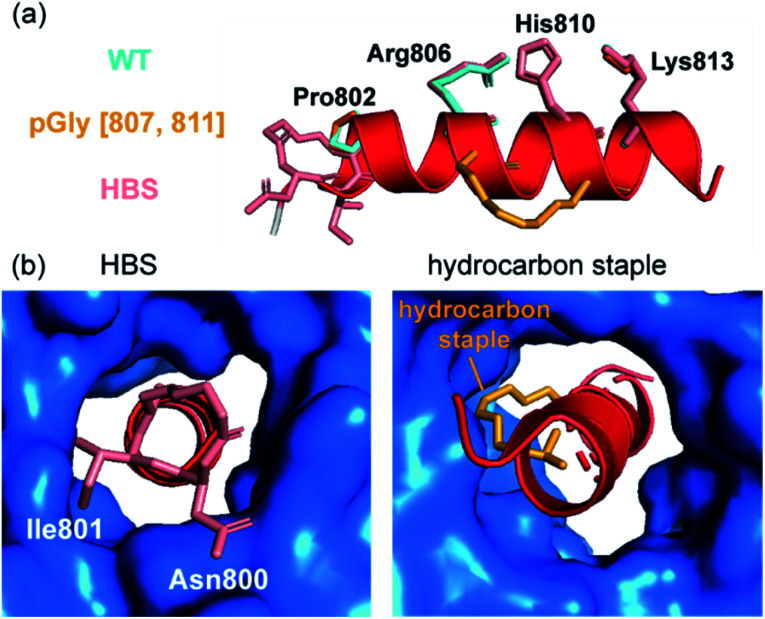
Constrained peptides as inhibitors of MyoA/MTIP interaction; (a) comparison of the helices formed by different peptides in complex with MTIP (blue): the native myoA tail (side chains in aquamarine, PDB: 4AOM), the hydrocarbon stapled peptides (side chains in orange, PDB: 4MZK) and HBS myo A in salmon (PDB: 4MZL); (b) the HBS motif is positioned at the N-terminus of the myoA peptide and the placement of the hydrocarbon staple in the center of the helix.^76^

A further approach used to constrain peptides in an α-helical conformation is the hydrogen bond surrogate (HBS),^18^ which provides a method to avoid unproductive interactions with protein surfaces. Douse *et al.* sought to address these challenges by comparing hydrocarbon stapling and HBS techniques for peptides targeting the interaction of *Plasmodium falciparum* myosin A (myoA) with myoA tail interacting protein (MTIP).^76^ Interestingly, the HBS myoA peptide showed comparable potency to that of wild-type myoA, as may have been expected given that the HBS constraint was intentionally positioned across a part of the myoA tail outside the congested MTIP binding site (Fig. 8a). However, upon introduction of a hydrocarbon staple, a decrease in potency was observed. Comparison of the crystal structures obtained for the constrained peptides to that of the wild-type revealed several key features; firstly, the fold of MTIP was maintained with the two domains clamped around the constrained myoA peptides and the hydrocarbon staple followed the same trajectory as the hydrophobic residues that were replaced (Fig. 8a). Since the HBS constraint was positioned at the N-terminus of the helix, steric clashes of the tether with the congested MTIP binding groove are avoided. In contrast, the placement of the hydrocarbon staple in the center of the helix requires the aliphatic chain to plug into the small hydrophobic core of the MTIP C-terminal domain, preventing optimal interaction between target protein and constrained peptide (Fig. 8b).

## 3 Determinants of cellular uptake of constrained peptides

Enhanced cellular uptake is a potential advantage of using constrained peptides as modulators of intracellular PPIs.^13^ Walensky and co-workers’ 2004 report that a hydrocarbon stapled α-helix BID BH3 peptide exhibited good cell permeability and *in vivo* inhibitory activity against human leukemia xenografts in mouse models,^54^ triggered extensive studies on the application of stapling to other intracellular targets including BCL-2 family interactions,^55,58^ and p53/*h*DM2.^77^ Although various stapled peptides have demonstrated promising activity against intracellular PPIs, attributing such activity solely to an on-target mechanism and correlating it with enhanced cell permeability is key. Czabotar and co-workers reported that a hydrocarbon stapled BIM-BH3-derived peptide, referred to as BimSAHB, exhibited weaker binding affinity to the BCL-2, BCL-x_L_, BCL-w and MCL-1 proteins in comparison to the unconstrained counterpart. In addition, cellular studies showed no evidence of BimSAHB-induced apoptosis in mouse embryonic fibroblasts (MEFs) or Jurkat cells, whilst a positive result was observed in cell lysates. Taken together, this suggests that the loss of activity of the stapled peptides in the cell models may result from both the reduction of binding affinity and poor cell permeability.^78^ Notably, BimSAHB has a different sequence from that of the stapled peptide (BIM SAHB_A1_) reported by Walensky and co-workers, which did show significant cytotoxicity and activity on caspase 3/7 activation in living cells (Table 1).^79^ BIM SAHB_A1_ was shown to enter cells *via* an endosomal mechanism rather than a membrane disruption mechanism, whereby the stapled peptide mainly localized to the mitochondria and multivesicular bodies of intact cells as demonstrated using electron microscopy and immunoelectron microscopy.^79^ In addition to the observations made by Czabotar and co-workers, the Walensky group noted the negative net charge of this alternative sequence – BimSAHB (BIM SAHB_A2_) – which may contribute to poor cell permeability; this would be consistent with a previous study conducted by the group, whereby the replacement of a single Arg residue with Asp had a marked negative impact on the cellular uptake of a 21-residue peptide.^80,81^ These results underscore the fact that minor sequence variants can confer distinct cell permeability profiles. Moreover, whilst much is known about entry mechanisms for specific stapled peptides not all questions are answered.^82^ Indeed, several studies have indicated constrained peptides have the capacity to induce cell lysis *via* cell membrane disruption, which is undesirable and likely to confer non-specific toxicity.^83–85^

**Table 1:** Comparative peptide sequences, biophysical properties, cellular effects and cellular permeabilities of BIM SAHB_A1_ and BIM SAHB_A2_

Peptide	Sequence^a^	α-Helicity (%)	Charge	Cellular effects^b^		Cell permeability^c^
				Viability (μM)	Caspase 3/7 activation	
BIM SAHB_A1_	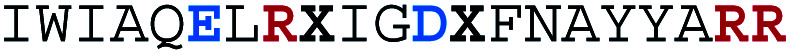	71	+1	∼5.5	+	+
BIM SAHB_A2_ (BimSAHB)	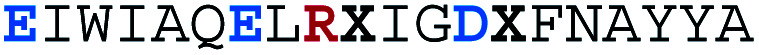	50	−2	>32	ND^d^	ND^d^

a Residues with negative charges are highlighted in blue; residues with positive charges are highlighted in red; X denotes residues participating in the formation of hydrocarbon staples.

b Results on OCI-AML3 cells are shown here.

c Cell permeability was verified by electron microscopy and immunoelectron microscopy; an endosomal mechanism of cell entry was confirmed.

d Not determined.

**Fig. 9:**
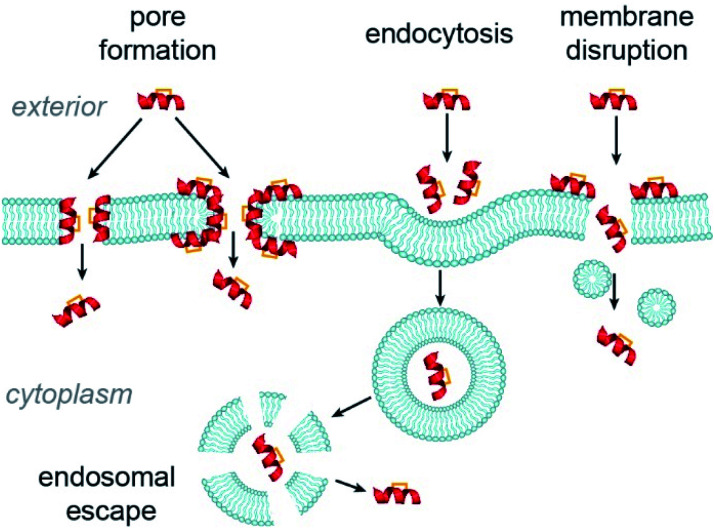
Possible internalization mechanisms of constrained peptides.

The major cellular internalization mechanism for constrained peptides is proposed to be *via* ATP-dependent endocytosis.^86^ Cell-penetrating peptides (CPPs) have been widely used in cellular studies because of their intrinsic cell permeability.^87^ The Verdine group compared the internalization mechanism of three wild-type CPPs and their hydrocarbon stapled counterparts.^86^ Contrary to the generally accepted ATP-dependent mechanism, they found that cell uptake of the stapled CPPs involved several mechanisms, among which ATP-independent endocytosis dominated. Nevertheless, the process by which constrained peptides escape the endosome into the cytosol remains less well studied.^82^ Other ATP-independent internalization mechanisms, such as pore-formation^88–90^ and cell membrane disruption,^91^ have also been observed in studies of CPPs (Fig. 9). As alluded to above, these mechanisms of cellular entry are also feasible for stapled peptides, for instance the Fairlie group reported that replacement of a lactam bridge with a hydrocarbon staple resulted in cell lysis.^85^ Li *et al.* devised a recombinase enhanced bimolecular luciferase complementation platform, termed ReBiL, to detect weak PPIs in living cells, which has proven powerful in studying the behaviour of stapled peptides.^92^ Three hydrocarbon stapled peptides, SAHp53-8,^63,77^ sMTide-02 ^66^ and ATSP-7041 ^64^ that show higher binding affinity to the *h*DM2 or *h*DMX *in vitro* than a known small molecule inhibitor, Nutlin-3a, were tested using this assay; each of the hydrocarbon stapled peptides showed little to no inhibitory activity in living cells. Under serum-free conditions the activities of the hydrocarbon stapled peptides in cells increased and cell membrane disruption was observed, whilst serum had no effect on the interactions in cell lysates, suggesting that the presence of serum inhibits the cellular entry of the peptides by preventing cell–membrane disruption.

Recently, further modification of constrained peptides has proven to offer an efficient approach to enhance cell permeability, *e.g.* by inserting cell-permeable motifs^93,94^ or self-assembly-promoting segments.^95^ However, incorporation of additional functionality may increase unwanted steric interactions or molecular recognition behaviour, increase synthetic complexity and may introduce further physicochemical liabilities into the peptide-based inhibitors. Therefore, constrained peptides lacking further modification represent more favourable candidates but their design – like peptide therapeutics in general – has largely been empirical.^96^ To rationally design cell-permeable stapled peptides, it is necessary to understand key principles for cell permeability.

Lin and co-workers reported that the cellular uptake of a designed MCL-1 BH3-derived constrained peptide was strongly correlated to the HPLC retention time.^97^ The relatively hydrophobic peptides showed enhanced cell permeability. Thereafter, the same group revealed that *N*-methylation of Ala in stapled peptides further improved cell permeability, suggesting that shielding polar groups can promote cellular uptake,^98^ noting that *N*-methylation is considered important for the cell permeability of numerous natural product and cyclic peptides.^82,96,99^

Fairlie and co-workers reported that point variants with hydrophobic amino acid residues exhibited improved activity in cell assays.^100^ A series of short lactam-bridged BAD BH3 domain sequence peptides with one or more incorporated hydrophobic residues were synthesized. Compared with the wild-type BAD peptide, the cyclic peptide (Ac-1Nal-Aib-Lys-Nle-Ala-Asp-Asp-Phe(Cl_2_)-NH_2_), incorporated norleucine (Nle), hydrophobic 1-naphthylalanine (1Nal), aminoisobutyric acid (Aib) and 3,4-dichlorophenylalanine (Phe(Cl_2_), (residues in italics denoting the site of the lactam bridge), showed 2-fold higher activity in a 3-(4,5-dimethylthiazol-2-yl)-2,5-diphenyltetrazolium bromide (MTT) assay, but exhibited 134-fold weaker binding affinity *in vitro* (Table 2) in comparison with the full-length native sequence. This implied that the enhanced cellular activity of the short peptide with hydrophobic residues was due to improved cellular uptake. Li and co-workers also demonstrated that a series of conformationally constrained CPPs with larger hydrophobic moments (HMs) exhibited stronger binding affinities with the cell membrane to promote endocytosis, highlighting the importance of tuning amphiphilicity.^101^

**Table 2:** Comparative sequences, binding affinities and activities on MTT assay of wild-type BAD peptide and cyclic peptide

Peptide	Sequence^a^	Binding affinity to BCL-x_L_ (μM)	IC_50_ on MTT assay (μM)
BAD	Ac-NLWAAQRYGRELRRMSDEFVDSFKK-NH_2_	0.052	62
Cyclic peptide	Ac-1Nal-Aib-*K*NleAD*D*-F(Cl_2_)-NH_2_	5.8	18

a Italic residues denote the positions participating in the formation of lactam staple.

In addition to sequence hydrophobicity, the hydrophobicity of the linker itself can have a significant effect on cellular uptake. The Li group investigated differences in cell permeability between peptides with different types of constraint, including the hydrocarbon staple, lactam bridge, triazole, vinyl sulfide, *m*-xylene and perfluoroaryl linkers, based on a peptide sequence targeting the estrogen receptor (ER) coactivator binding site.^102^ In this study, stapled peptides with more hydrophobic linkers, *e.g.* hydrocarbon and perfluoroaryl bridges, showed 2-10-fold higher cell permeability than linear or hydrophilic-linker derivatives in different cell lines (Table 3). The cell permeability of the stapled peptides with different staples was found to correlate strongly with the hydrophobicity using flow cytometry analysis and reverse-phase liquid chromatography.

**Table 3:** Comparative α-helicity, hydrophobicity and cell permeability orders of the model peptide targeting the ER coactivator^a^

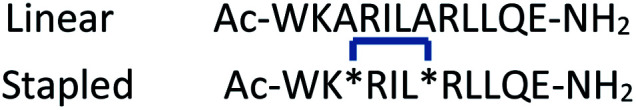			
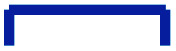	Helicity order	Hydrophobicity order	Cell permeability order^b^
Hydrocarbon	1	1	4
Lactam	1	6	6
Triazole	3	5	5
Vinyl sulfide	4	4	3
*m*-Xylene	5	3	2
Perfluoroaryl	7	2	1
None (linear)	6	6	7

a The same numbers denote the two stapled peptide showed similar specific properties.

b The cell-permeabilities were detected using FAM-labelled stapled peptides in T47D, MCF-7, Hela, HEK293T cell lines *via* confocal microscopy, which showed concordant results in this study.

There have also been analyses linking the impact of peptide α-helicity on its cell uptake. The Verdine group systematically investigated the influence of the absolute configuration of building blocks for hydrocarbon stapling on α-helicity and cellular uptake, based on a model sequence from RNase A.^103^ The replacement of an (*S*,*S*)-configured pair of unnatural amino acids to an (*R*,*R*)-configured pair resulted in loss of α-helicity and a decrease in cellular uptake. Recently, Li and co-workers incorporated different substituents, including hydrogen, methyl, ethyl, isopropyl, phenyl and benzyl groups, into a peptide targeting estrogen receptor α (ER-α) which was stapled using thiol–ene chemistry to create an in-tether chiral center.^104^ These stapled peptides showed different α-helicities although they were stapled in identical sequence positions. Additionally, the cell permeability of stapled variants was found to strongly correlate with their α-helicities, except for the non-substituted stapled peptide, which showed the highest α-helicity but the lowest cellular uptake. A related study from Li and co-workers unambiguously established the relevance of helical content on cell uptake by comparing uptake of different in-tether epimers.^105^ A 2017 study by Ulrich and co-workers also indicated α-helicity was beneficial for cell uptake. A series of lactam stapled CPPs were shown to cause less membrane leakage/lysis than the unstapled analogues. Importantly here α-helix content of the peptides when bound to lipid membranes, rather than in solution, correlated with cell uptake.^106^ An important study published by Futaki and co-workers however highlighted hydrophobicity as more important than helicity.^107^ A comparative series of six previously described peptides (targeting the p53/DM2 interaction) and their unstapled parent were reported to enter cells *via* endocytosis. The unstapled peptides with greater hydrophobicity were shown to have greater cell uptake. Non-helical cell-permeable stapled peptides have also been reported.^108^ Spring and co-workers reported a series of double-triazole stapled peptides based on a nuclear localization signal (NLS) sequence targeting the interaction between HNF1β and importin α1, some of which penetrated the cell membrane.^108^

Net charge has also been shown to play an important role in defining the cellular uptake properties of stapled peptides (see above). In Verdine's study, increasing net charge from −1 to +5 was found to improve the cellular uptake of both hydrocarbon stapled and stitched peptides^86^ (a unique form of double stapled peptides with two contiguous staples bridging three residues^109^). However, the constrained peptides with net positive charge over +7 showed a significant decrease in cell permeability.

**Fig. 10:**
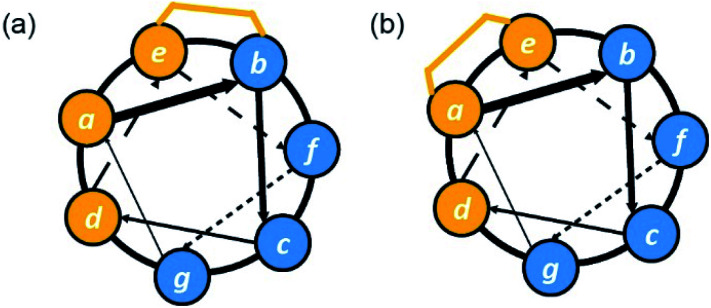
Helical wheel diagrams illustrating subtle staple placement; (a) at the boundary between hydrophobic and hydrophilic surfaces which is likely to result in improved uptake due to expansion of hydrophobic surface area; (b) to form a contiguous hydrophobic surface which is likely to confer superior uptake. Hydrophobic residues and staples are shown in orange, and hydrophilic and positively charged residues are shown in blue.

The cellular uptake of constrained peptides therefore clearly depends on several driving factors, *e.g.* hydrophobicity, α-helicity, net charge and the presence of serum. However, cellular studies have usually focused on one of the key factors independently. To provide comprehensive guidelines for design of cell-permeable constrained peptides, Walensky and co-workers developed a high-throughput fluorescence microscopy approach to measure the cellular uptake of hydrocarbon stapled peptides with N-terminal FITC labels.^84^ In this work, the group systematically tested three classes of hydrocarbon stapled peptides, including hydrocarbon stapled BIM BH3 helices with different stapling positions, or diverse single-residue variants, and hydrocarbon stapled peptides based on another sequence (RAS binding SOS1). Unbiased quantitative analytical protocols, used to clarify key biophysical factors influencing the cellular uptake of these peptides, revealed that overall hydrophobicity, net charge, α-helicity, and staple placement are key determinants of cell penetration. The overall hydrophobicity of the stapled peptides, which was determined by HPLC retention time, correlates with cellular uptake. The stapled peptides with high, but not overwhelming hydrophobicity, showed optimal cell permeability. The position of a staple also plays an important role in modulation of cellular uptake of the stapled peptides. Subtle changes in position of a hydrocarbon staple between a hydrophobic surface and a hydrophilic surface were found to improve the cellular uptake of the stapled peptides due to the expansion of the hydrophobic surface (Fig. 10).^84^ Furthermore, α-helicity and isoelectric point (pI) were identified as influences on cell permeability in Walensky's study.^84^ In combination with high α-helicity (61–86%), the relatively hydrophobic stapled peptides showed excellent cell permeability. However, excessive hydrophobicity coupled with highly acidic pI was shown to confer a high tendency to induce cell lysis.

Recently, the Fairlie group tested the cell permeability of a series of FITC-labelled stapled peptides quantitively *via* flow cytometry in living cells without serum.^85^ A cell-penetrating peptide, TAT, was selected as a control for 100% of cell entry. The cell permeability was found to be correlated to the connected hydrophobic surface area (cHSA) and the hydrophobic moment (*μ*_H_), which were determined computationally. The constrained peptides lacking amphipathic structures were not cell-permeable. In addition, amphipathic peptides lacking positively charged residues showed poor cell permeability, demonstrating that amphipathicity itself is not sufficient to promote cellular uptake. Notably, however, the presence of both a hydrophobic patch (consisting of a hydrophobic bridge and hydrophobic residues) and a contiguous charged surface significantly enhanced the cell permeability of short lactam- and hydrocarbon-bridged peptides (Fig. 10).^85^ In this study, the group also found that the insertion of a hydrocarbon staple is markedly more likely to induce cell lysis than its lactam-bridged counterpart.

Finally, peptides that consist of all-d-amino acids generally have high proteolytic stability but poor cell permeability.^110^ Recently however, Kannan *et al.* developed a series of hydrocarbon stapled and stitched, all-d-amino acid peptides to target the p53/MDM2 PPI, some of which exhibited improved cell permeability.^111^ The group utilised a lactate dehydrogenase (LDH) release assay to assess the membrane integrity and a counterscreen assay to validate intracellular p53 engagement. While the most potent stapled peptides in this study, dPMI-δ (5–12), and stitched peptide, dPMI-δ (1–5–12), led to p53 activation and exhibited no membrane disruption, some of the stapled peptides caused cellular leakage. It is likely that the increased cell permeability of the stitched peptide results from the increased hydrophobicity and conformational rigidity. However, a double-stapled peptide, dPMI-δ (1–5, 9–12), exhibited stronger binding to MDM2 in an FP assay but poorer cellular activity in comparison to the stitched peptide, suggesting a possible loss of cell permeability despite increased hydrophobicity and structural stability. Thus, these somewhat contradictory results for two peptides with increased hydrophobicity point to more subtle effects of hydrophobicity on cell permeability.

Overall, these examples indicate a positive effect on the cell uptake behaviour arising from introduction of a constraint, emphasize the complex sequence/cell permeability space that must be navigated to identify peptides with optimal permeability and provide empirical guidelines to do so. Overall hydrophobicity, net positive charge, α-helicity, and contiguous charged and hydrophobic surfaces should be considered as appropriate starting points. In addition, the cell type is undoubtedly important and systematic studies will aid in refining these guidelines; for instance recent studies have shown better uptake observed for conformationally stabilized peptides in cancer cells with upregulated macropinocytosis.^112^ It is noteworthy that a significant number of these studies make use of fluorescently labelled peptides which may behave differently to the parent sequence as a consequence of the fluorescent reporter. Making use of novel higher-throughput assays *e.g.* the recently reported chloroalkane penetration assay,^113^ methods that can rapidly generate diverse and/or libraries of constrained peptides^114,115^ and machine learning^116^ holds significant potential in this respect. Already, using combinatorial approaches to constraining peptides targeting p53/DM2, significant differences in bioactivity have been observed with endosomal/lysosomal entrapment playing an important role and nonspecific toxicity arising from a combination of staple linker and peptide sequence, not necessarily either in isolation.^117^ Similarly, the wealth of systematic and physicochemical studies on cell-penetrating peptides and (natural product) cyclic peptides^118–121^ offer much insight that can be applied to designed constrained peptides targeting PPIs in future.

## 4 Conclusions

In the past two decades, development of synthetic constraining methods has prompted the rapid discovery of peptides to perturb traditionally “undruggable” PPIs. Compared to wild-type peptides, constrained peptides are generally expected to have improved binding affinities, similar binding behaviour and enhanced cellular uptake. Although not discussed in detail here, we note also that improved proteolytic stability is an advantage of constrained peptides, and the reader is directed to numerous reports that describe this well studied property.^13,54^ Recent findings highlight hidden complexities which we have summarized here to illustrate how the blueprint for rational design of constrained peptides is evolving. Binding mechanism and thermodynamics should be considered alongside the nature of any direct interaction between protein and constraint, subtle structural influences on bound/unbound conformation and the complex sequence-structure space determining cell uptake. Other features of constrained peptides, *e.g.* distribution, metabolism, clearance, immunogenicity and other PK/PD properties have yet to be systematically studied. Nonetheless, significant barriers to the use of linear-peptide ligands have been surpassed using this class of peptides, leading to candidates that have entered clinical trials.^11,122^ The development of constrained peptides to target an increasingly broad range of targets^123–125^ alongside their integration into proteolysis targeting chimeras^126^ ensures they can confidently be predicted to remain an important class of compounds in chemical biology and drug discovery research.

## Author contributions

X. W. and A. J. W. conceived the scope and focus of the review. The selection of material was made by H. W., R. D. and P. Z. who wrote the manuscript with contributions from all authors. M. W., A. J. W. and X. W. edited the manuscript into its final form.

## Conflicts of interest

There are no conflicts to declare.
